# Potential Roles of CCR5^+^ CCR6^+^ Dendritic Cells Induced by Nasal Ovalbumin plus Flt3 Ligand Expressing Adenovirus for Mucosal IgA Responses

**DOI:** 10.1371/journal.pone.0060453

**Published:** 2013-04-02

**Authors:** Yoshiko Fukuyama, Daisuke Tokuhara, Shinichi Sekine, Kazuyoshi Aso, Kosuke Kataoka, Julia Davydova, Masato Yamamoto, Rebekah S. Gilbert, Yuka Tokuhara, Keiko Fujihashi, Jun Kunisawa, Yoshikazu Yuki, Hiroshi Kiyono, Jerry R. McGhee, Kohtaro Fujihashi

**Affiliations:** 1 Departments of Pediatric Dentistry and Microbiology, The Immunobiology Vaccine Center, The Institute of Oral Health Research, The University of Alabama at Birmingham, Birmingham, Alabama, United States of America; 2 Division of Mucosal Immunology, Institute of Medical Science, University of Tokyo, Tokyo, Japan; 3 Department of Preventive Dentistry, Graduate School of Dentistry, Osaka University, Osaka, Japan; 4 Department of Preventive Dentistry, Institute of Health Biosciences, The University of Tokushima Graduate School, Tokushima, Japan; 5 Department of Surgery, University of Minnesota, Minneapolis, Minnesota, United States of America; 6 Laboratory of Vaccine Materials, National Institute of Biomedical Innovation, Osaka, Japan; Universidade Federal do Rio de Janeiro, Brazil

## Abstract

We assessed the role of CCR5^+^/CCR6^+^/CD11b^+^/CD11c^+^ dendritic cells (DCs) for induction of ovalbumin (OVA)-specific antibody (Ab) responses following mucosal immunization. Mice given nasal OVA plus an adenovirus expressing Flt3 ligand (Ad-FL) showed early expansion of CCR5^+^/CCR6^+^/CD11b^+^/CD11c^+^ DCs in nasopharyngeal-associated lymphoid tissue (NALT) and cervical lymph nodes (CLNs). Subsequently, this DC subset became resident in submandibular glands (SMGs) and nasal passages (NPs) in response to high levels of CCR-ligands produced in these tissues. CD11b^+^/CD11c^+^ DCs were markedly decreased in both CCR5^−/−^ and CCR6^−/−^ mice. Chimera mice reconstituted with bone marrow cells from CD11c-diphtheria toxin receptor (CD11c-DTR) and CCR5^−/−^ or CD11c-DTR and CCR6^−/−^ mice given nasal OVA plus Ad-FL had elevated plasma IgG, but reduced IgA as well as low anti-OVA secretory IgA (SIgA )Ab responses in saliva and nasal washes. These results suggest that CCR5^+^CCR6^+^ DCs play an important role in the induction of Ag-specific SIgA Ab responses.

## Introduction

Nasal delivery of antigen (Ag) given together with a mucosal adjuvant has emerged as an effective way to induce both peripheral and mucosal immunity, including secretory IgA (SIgA) antibody (Ab) responses. In this regard, nasopharyngeal-associated lymphoid tissue (NALT) contains all of the immune cells required for the induction and regulation of the mucosal immune response to Ags delivered into the nasal cavity [1], [2]. Our previous studies showed that nasal administration of a naked cDNA plasmid expressing Flt3 ligand cDNA (pFL) enhanced CD4-positive (CD4^+^) Th2- type cytokine-mediated mucosal immunity and increased lymphoid-type dendritic cell (DC) numbers [3]. Of interest, mucosal delivery of Flt3 ligand cDNA via a recombinant adenovirus (Ad-FL) also exhibited mucosal adjuvanticity through stimulation of NALT DCs [4]. Nasal delivery of ovalbumin (OVA) plus Ad-FL induced CD4^+^ Th1- and Th2-type responses as well as significant plasma IgG and IgA and SIgA anti-OVA Abs in external secretions. Further, the numbers of CD11b^+^ CD11c^+^ DCs expressing high levels of co-stimulatory molecules were preferentially induced. These CD11b^+^ CD11c^+^ DCs migrated from the NALT to mucosal effector lymphoid tissues via the cervical lymph nodes (CLNs) [4]. Based upon these findings, we thought it important to determine how chemokines and their receptors affect migration of this DC subset into mucosal effector tissues for the induction of SIgA Ab responses. To pursue this, we assessed chemokine receptors expressed by DCs in both mucosal inductive (NALT) and effector [submandibular glands (SMGs) and nasal passages (NPs)] sites of mice following nasal delivery of OVA and Ad-FL as mucosal adjuvant.

It has been shown that both C-C chemokine receptor (CCR) 6 and CCR7 play important roles in DC relocation and migration both within and between mucosal lymphoid tissues [5], [6], [7], [8], [9]. Thus, immature DCs in Peyer’s patches (PPs) express CCR6 which controls their movement into the subepithelial dome area [6]. These DCs express CCR7 after Ag uptake, undergo maturation and then relocate to the T cell areas of PPs. Further, CCR6^+^ DCs in the small intestinal lamina propria migrate into mesenteric lymph nodes (MLNs) after capturing luminal Ags [10]. A second major chemokine receptor CCR1 is expressed by CD11b^+^ DCs in the dome region of PPs [11]. The epithelial cells covering PPs produce the CCR1 ligand CCL9 which regulates CD11b^+^ CD11c^+^ DC recruitment [11]. Antigen uptake in the lungs also leads to DC recruitment. In this regard, knockout of CCR2 resulted in impaired pulmonary DC activation with diminished inflammation [12]. Recent studies have shown that CCR7 plays a key role in migration of local DCs into CLNs following sublingual immunization [13].

Taken together, these studies indicate that it is important to characterize the chemokine receptor expression by Ad-FL-induced CD11b^+^ DCs in NALT which ultimately leads to the induction of Ag-specific immune responses.

## Materials and Methods

### Mice

Young adult 6- to 8- week (wk) old C57BL/6 mice were purchased from the Frederick Cancer Research Facility (National Cancer Institute, NIH, Frederick, MD). CCR5^−/−^, CCR6^−/−^ and CD11c-DTR mice on a C57BL/6 background were obtained from The Jackson Laboratory (Bar Harbor, ME). Upon arrival, all mice were transferred to microisolators, maintained in horizontal laminar flow cabinets, and provided sterile food and water in a specific-pathogen-free animal facility at the University of Alabama at Birmingham (UAB) Immunobiology Vaccine Center (IVC). All mice used in these experiments were free of bacterial and viral pathogens. All experiments involving mice were performed in accordance with both NIH and the UAB Institutional Animal Care and Use Committee (IACUC) guidelines. The UAB IACUC gave specific approval for all procedures involving mice; animal protocol number 110908212.

### Preparation of Chimera Mice by Bone Marrow Transplantation

C57BL/6 mice were irradiated (1000 rads), and injected intravenously with a mixture of bone marrow cells from CD11c-DTR and C57BL/6 (CD11c-DTR/C57BL/6), CD11c-DTR and CCR5^−/−^ (CD11c-DTR/CCR5^−/−^), or CD11c-DTR and CCR6^−/−^ (CD11c-DTR/CCR6^−/−^) mice 6 h after irradiation (Figure S1). Six weeks after bone marrow transplantation, recipient mice were nasally immunized three times at weekly intervals with OVA plus Ad-FL as adjuvant. Diphtheria toxin (DT) (100 ng/mouse; Sigma-Aldrich, St. Louis, MO) was injected into CD11c-DTR/C57BL/6 chimera, CD11c-DTR/CCR5^−/−^ chimera and CD11c-DTR/CCR6^−/−^ chimera mice as well as CD11c-DTR mice via the intraperitoneal route 6 h before each nasal immunization.

### Preparation of the Adenovirus Vector

Replication-incompetent adenovirus vectors expressing FL (Ad-FL) and firefly luciferase (Ad-Luc), respectively, were constructed through homologous recombination in *Escherichia coli* using the AdEasy system [4]. The vectors used in our experiments contained transgene cassettes driven by the human CMV promoter placed in the E1-deleted region of an adenoviral vector backbone. Thus, the recombinant Ad-FL was constructed by inserting the murine FL cDNA into an early region (E1). Expression of cDNA was driven by the human CMV immediate gene promoter and terminated by the polyadenylation sequence, poly (A), of SV40. The viruses were propagated in the Ad-packaging cell line, human embryonic kidney (HEK) 293 cells (Microbix Biosystems), and purified by twice repeating a CsCl density gradient centrifugation step followed by dialysis against PBS with 10% glycerol. The Ad vectors were titrated by plaque assay and stored at −80°Celsius (C) until used.

### Nasal Immunization and Sample Collection

Mice were nasally immunized three times at weekly intervals with 3 µl per nostril of PBS containing 1×10^8^ PFU of Ad-FL and 100 µg of OVA (Sigma-Aldrich). Plasma, saliva and nasal washes (NWs) were collected on day 21. Saliva was obtained from mice following intraperitoneal injection of 100 µg of pilocarpine hydrochloride (Sigma-Aldrich). NWs were collected by gently flushing the NPs with 1 ml of Ringer’s lactate (Abbot Laboratories, North Chicago, IL).

### OVA-specific ELISA

OVA-specific Abs in plasma, saliva and NWs were determined by ELISA as previously described [3], [4], [14]. Briefly, 96-well Falcon microtest assay plates (BD Biosciences, San Jose, CA) were coated with 1 mg/ml of OVA in PBS. After blocking with 1% bovine serum albumin (BSA) (Sigma-Aldrich) in PBS, 2-fold serial dilutions of samples were added and incubated overnight at 4°C. Horse radish peroxidase (HRP)-labeled goat anti-mouse µ, γ or α heavy chain-specific Abs (Southern Biotechnology Associates, Birmingham, AL) were added to individual wells. The color reaction was developed for 15 min at room temperature with 100 µl of 1.1 mM 2, 2′-azino bis (3-ethylbenz-thiazoline-6-sulfonic acid) (ABTS; EMD Biosciences, La Jolla, CA). Endpoint titers were expressed as the reciprocal log_2_ of the last dilution that gave an optical density at 415 nm of 0.1 greater than background.

### OVA-specific ELISPOT

Mononuclear cells from the spleen, NPs and SMGs were isolated as described previously [4], [14], [15], [16], [17]. Cells were subjected to an ELISPOT assay to determine the numbers and isotype of OVA-specific Ab-forming cells (AFCs). Briefly, 96-well nitrocellulose plates (Millititer HA; Millipore, Billerica, MA) were coated with 1 mg/ml of OVA for analysis of anti-OVA-specific AFCs. The numbers and isotype of OVA-specific AFCs were quantified using a CTL-ImmunoSpot® analyzer (Cellular Technology Limited, Shaker Heights, OH).

### Flow Cytometry Analysis

To characterize the phenotype of DCs, mononuclear cells (0.2–1×10^6^) from spleens, CLNs, NALT, NPs and SMGs were isolated one week after the last immunization. The cells were stained with FITC-conjugated anti-CD11b, PE-labeled anti-CCR5, allophycocyanin-tagged anti-CCR6 or -CCR7 and biotinylated anti-CD11c mAbs followed by PerCP-Cy5.5-streptavidin (BD Biosciences). In order to confirm CD11c staining for DCs, cells were incubated with an Ab cocktail consisting of FITC-conjugated anti-CD3 (145-2C11), -CD49b (DX5), -NK1.1 (PK136) and -Ig (goat anti-mouse), and biotinylated anti-CD11c mAb. In some experiments, mononuclear cells were incubated with FITC-conjugated anti-CD11b, PE-labeled anti-CD11c, allophycocyanin-tagged anti-B220 and biotinylated anti-CD8 (BD Biosciences) mAbs followed by PerCP-Cy5.5-streptavidin. These samples were subjected to flow cytometry (FACS Calibur; BD Biosciences) for cell subset analysis.

### CCL3, CCL4, CCL5 and CCL20 Production

Mononuclear cells from NALT, NPs and SMGs were isolated three days after the second immunization (day 10) or one week after the last immunization (day 21), and cultured for 5 days. The supernatants from individual wells were then subjected to the respective chemokine-specific ELISA kits (R&D Systems), according to the manufacturer’s instructions.

### Statistical Analysis

The results are presented as the mean ± one standard error of the mean (SEM). Groups of C57BL/6 mice nasally immunized with OVA plus Ad-FL were compared with mice nasally immunized with OVA and Ad-Luc using an unpaired Mann-Whitney *U* test with Statview software (Abacus Concepts, Cary, NC) designed for Macintosh computers. In some experiments, CCR5^−/−^ or CCR6^−/−^ mouse groups nasally immunized with OVA plus Ad-FL were compared with identically immunized, normal C57BL/6 mice. DT treated, CD11c-DTR/CCR5^−/−^ or CD11c-DTR/CCR6^−/−^ mouse groups given nasal OVA plus Ad-FL were compared with identically immunized, DT treated, CD11c-DTR/C57BL/6 chimera mice. Values of *p*<0.05 or *p*<0.01 were considered significant.

## Results

### Nasal OVA and Ad-FL Induces Increased Numbers of CCR5^+^ and CCR6,^+^ CD11b^+^ DC Subsets in Nasal-oral Lymphoid Tissues

**Figure 1:**
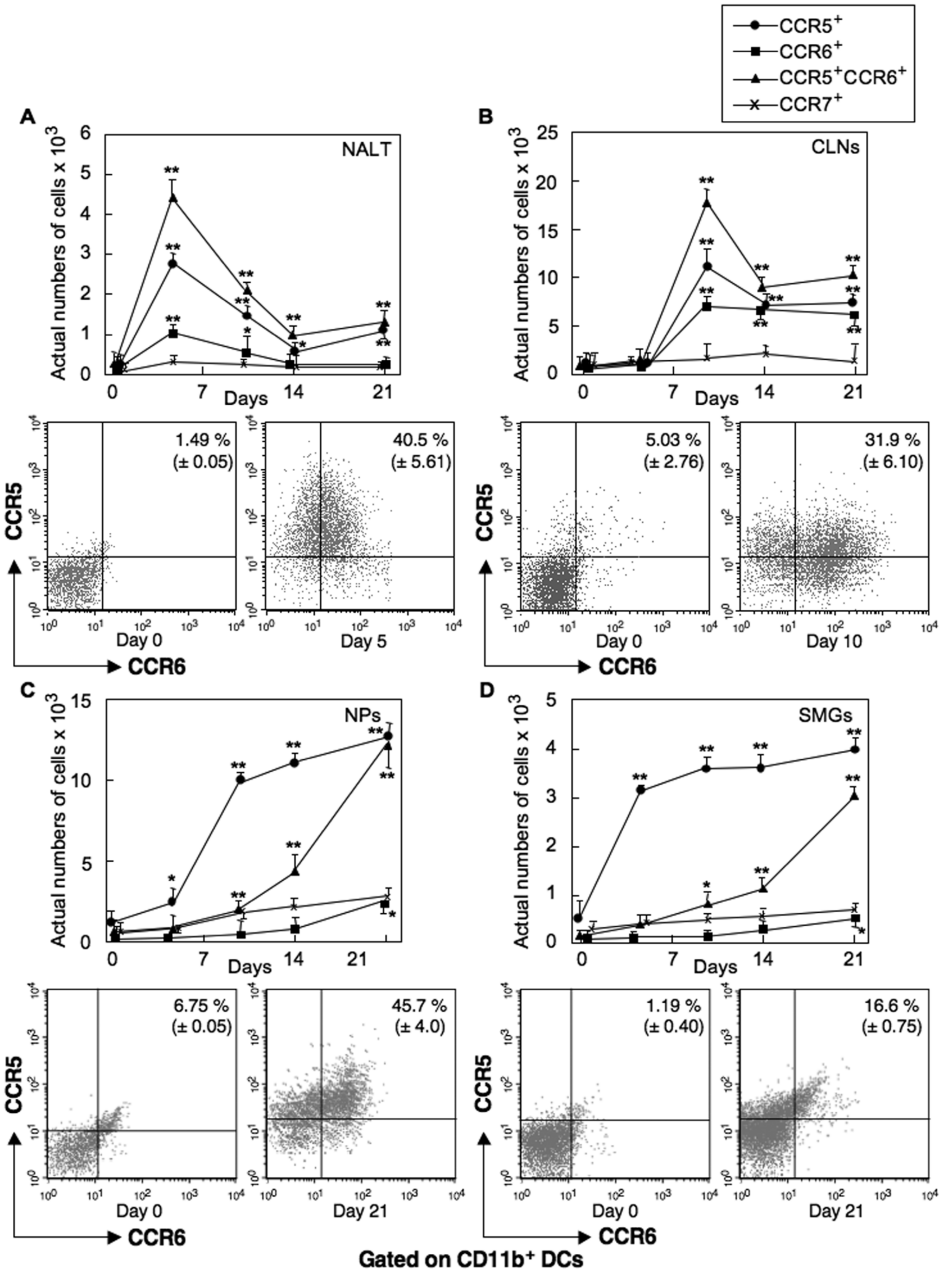
Kinetics of chemokine receptor expression by CD11b^+^ DCs. Mice were nasally immunized weekly for three consecutive weeks with OVA plus Ad-FL (A-D). Five, 10, 14 and 21 days after the initial immunization, mononuclear cells (MNCs) from NALT (A), CLNs (B), NPs (C), and SMGs (D) were stained with FITC-conjugated anti-CD11b, PE-labeled anti-CCR5, allophycocyanin-tagged anti-CCR6 or -CCR7 and biotinylated anti-CD11c mAbs followed by PerCP-Cy5.5-streptavidin. CD11c^+^ and CD11b^+^ cells were gated and their CCR expression was analyzed by FACSCalibur®. The values shown are the mean of the actual numbers of cells ± SEM taken from five separate experiments (two samples/experiment) with a total of 10 samples in each experimental group. * *p*<0.05, ** *p*<0.01 when compared with day 0.

Our previous studies showed that CD11b^+^ DCs in mucosal effector tissues originate from NALT after nasal delivery of Ag and Ad-FL. This CD11b^+^ DC subset was shown to express high levels of CD11c molecules but were negative for F4/80, CD3, CD49b, NK1.1 and Ig. Our initial experiments in the present study assessed the frequencies of CD11b^+^ DCs expressing the chemokine receptors CCR5, CCR6 and CCR7 in mucosal and peripheral lymphoid tissues of mice given nasal OVA plus Ad-FL as mucosal adjuvant. Early expansion of CCR5^+^ and CCR6^+^ CD11b^+^ DC populations were seen in NALT and CLNs of C57BL/6 mice (Figure 1A and 1B). In this regard, the highest numbers of CCR5^+^ CCR6^+^ CD11b^+^ DCs were noted in NALT five days after the first nasal immunization with OVA plus Ad-FL (Figure 1A), and the numbers of CCR5^+^, CCR6^+^ CD11b^+^ DCs in CLNs peaked three days after the second immunization (day 10) (Figure 1B). CCR5^+^, CCR6^−^ CD11b^+^ DCs showed a similar pattern of kinetics in NALT and CLNs (Figure 1A and 1B). Of interest, CCR5^+^, CCR6^+^ CD11b^+^ DC subsets in NPs and SMGs developed later and increased in a time-dependent manner (Figure 1C and 1D). In contrast, CCR5^+^, CCR6^−^ CD11b^+^ DCs showed an earlier expansion in NPs and SMGs (Figure 1C and 1D). Of importance, although CCR7-expressing CD11b^+^ DC subsets were seen in NALT, CLNs, NPs or SMGs, the frequency of this DC subset did not increase significantly (Figure 1A–D). These results show that nasal OVA and Ad-FL induces increased numbers of CCR5^+^ and CCR6^+^ CD11b^+^ DC subsets in NALT, and suggest the possible migration of these DC subsets into the effector lymphoid tissues through CLNs and the systemic circulation.

### CCL3, CCL4, CCL5 and CCL20 Expression in Mice Nasally-immunized with OVA Plus Ad-FL

**Figure 2:**
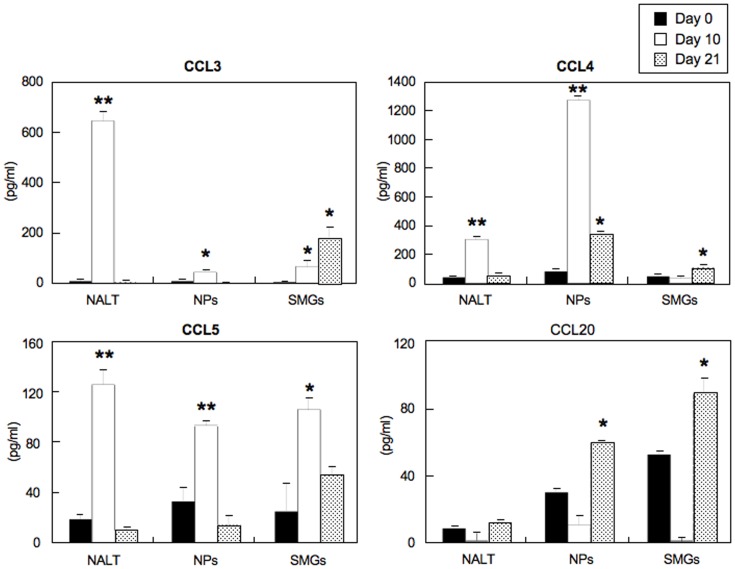
CCL3, CCL4, CCL5 and CCL20 synthesis in various mucosal tissues. Mice were nasally immunized weekly for either two or three consecutive weeks with OVA plus Ad-FL. Three days after the second immunization (day 10) or one week after the last immunization (day 21), MNCs were taken from NALT, NPs and SMGs, and cultured *in vitro* for 5 days. Culture supernatants were harvested after 5 days of incubation and analyzed for the respective chemokine by ELISA. The values shown are the mean ± SEM taken from five separate experiments with a total of 25 mice in each experimental group. * *p*<0.05, ** *p*<0.01 when compared with naïve mice (day 0).

We next assessed expression levels of CCL3, CCL4 and CCL5 (all ligands for CCR5) and CCL20 (ligand for CCR6) in both nasal-oral inductive and effector tissues of mice nasally immunized with OVA plus Ad-FL. Increased levels of all three CCR5 ligands were seen in the NALT of mice nasally immunized with OVA plus Ad-FL at day 10; however, these responses had returned to basal levels by day 21 (Figure 2). A similar pattern of CCL3 and CCL5 production was noted in the NPs (Figure 2). Of importance, NPs contained increased levels of both CCR5- and CCR6-ligands (CCL4 and CCL20) at day 21 (Figure 2). The SMGs revealed elevated levels of CCL3 and CCL5 at day 10 when compared with chemokine levels at day 0 (Figure 2). Further, CCL3, CCL4 and CCL20 production was significantly upregulated in the SMGs at day 21 after nasal immunization (Figure 2). These results suggest that CCL3, CCL4 and CCL5 as well as CCL20 play important roles in the recruitment of CCR5- and CCR6-expressing DCs for the induction of mucosal immune responses.

### Decreased Numbers of DCs Occur in Nasally Immunized CCR5 Gene-deficient (CCR5^−/−^) and CCR6 Gene-deficient (CCR6^−/−^) Mice

**Figure 3:**
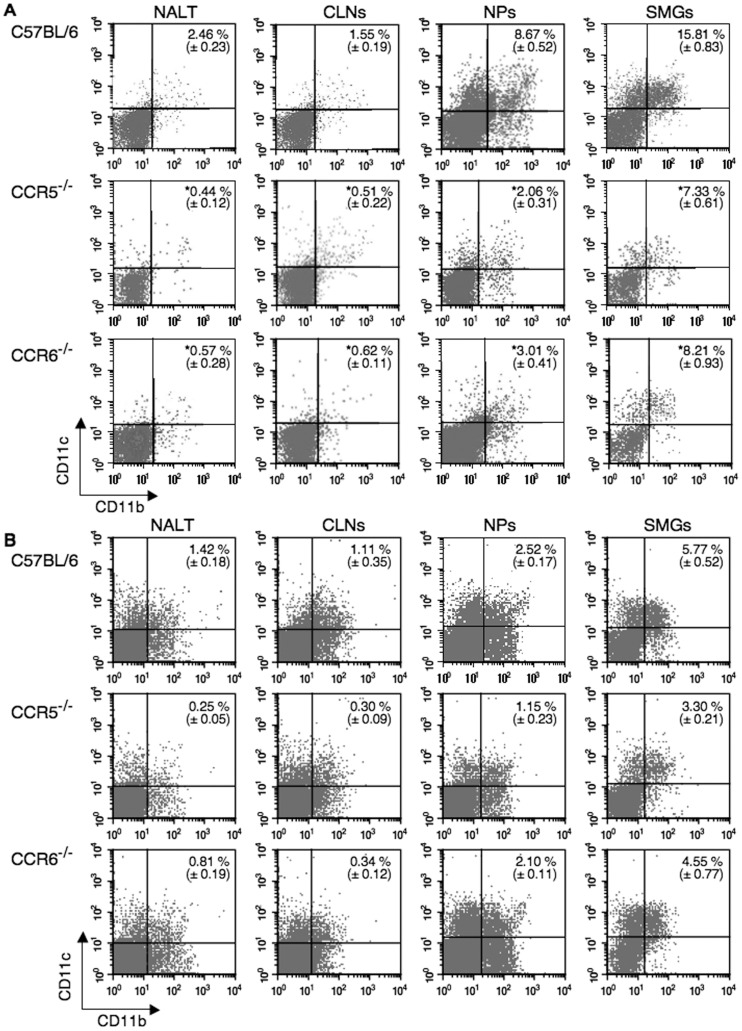
Comparison of the frequency of CD11b^+^ DCs in mucosal and peripheral lymphoid tissues of normal, CCR5^−/−^ and CCR6^−/−^ C57BL/6 mice. Mice were nasally immunized weekly for three consecutive weeks with OVA plus Ad-FL. (A) One week after the last immunization or (B) three days after the second immunization (day 10), MNCs were taken from NALT, CLNs, NPs and SMGs, and stained with FITC-conjugated anti-CD11b and PE-labeled anti-CD11c. All samples were subjected to flow cytometry analysis with a FACSCalibur®. After lymphocyte gating, (A) 10,000 cells were assessed whereas (B) 50,000 cells were taken for the analyses since the frequencies of CD11b^+^ DCs at day 10 samples were limited. The results represent the mean values ± SEM from three separate experiments with a total of 15 mice in each experimental group and are taken from three separate experiments. The profiles represent typical results and are taken from one of three separate experiments. *p<0.05 when compared with normal C57BL/6 mice.

We next investigated the frequency of DCs in various mucosal inductive and effector tissues of CCR5^−/−^ or CCR6^−/−^ mice given nasal OVA plus Ad-FL. The numbers of CD11c^+^ DCs were decreased in NALT, CLNs, NPs, SMGs and spleens of both CCR5^−/−^ and CCR6^−/−^ mice given nasal OVA plus Ad-FL seven days after the last immunization when compared with identically immunized C57BL/6 mice (Table 1). Importantly, the numbers of CD11b^+^CD11c^+^ DCs were significantly decreased in NALT, CLNs, NPs and SMGs of both CCR5^−/−^ and CCR6^−/−^ mice given nasal OVA plus Ad-FL on day 21 when compared with identically immunized C57BL/6 mice (Table 1 and Figure 3A). Interestingly, at an earlier time point (day 10), reduced numbers of CD11b^+^CD11c^+^ DCs were seen in the NALT and CLNs of CCR5^−/−^ and CCR6^−/−^ mice. Of interest, CCR5^−/−^ but not CCR6^−/−^ mice showed significantly reduced numbers of CD11b^+^CD11c^+^ DCs in the NPs and SMGs at day 10 (Figure 3B). Although reduced frequencies of CD8^+^ or B220^+^ CD11c^+^ DCs were also seen in CCR5^−/−^ and CCR6^−/−^ mice, the levels of reduction were not as significant as that seen in the CD11b population (Table 1). These results suggest that both CCR5 and CCR6 play key roles in the increase of CD11b^+^ DCs in mucosal lymphoid tissues.

**Table 1:** Comparison of the subpopulation of CD11c^+^ DCs in mucosal and peripheral lymphoid tissues of mice given nasal OVA plus Ad-FL^a^.

Tissue	Mice	Actual Numbers of Cells in Total Lymphocytes (x 10^4^)	Actual Numbers of DC subsets inTotal Lymphocytes (x 10^4^)		
		CD11c b, d	CD8^+^ CD11c^+^ c, d	CD11b^+^ CD11c^+^ c, d	B220^+^ CD11c^+^ c, d
NALT	Naïve	2.55 (±1.73)	0.26 (±0.02)	0.62 (±0.03)	1.52 (±0.03)
	C57BL/6	8.10 (±1.49)	0.77 (±0.01)	3.25 (±0.02)	4.40 (±0.03)
	CCR5^−/−^	2.89 (±1.45) **	0.27 (±0.04) **	0.91 (±0.05) **	1.71 (±0.02) **
	CCR6^−/−^	2.75 (±1.07) **	0.27 (±0.02) **	0.93 (±0.03) **	1.54 (±0.04) **
CLNs	Naïve	41 (±8.1)	8.7 (±0.19)	10.6 (±0.21)	16.6 (±0.18)
	C57BL/6	170 (±35.5)	34.9 (±0.89)	66.7 (±1.42)	67.2 (±0.67)
	CCR5^−/−^	86 (±31.9) **	18.0 (±1.89) **	18.5 (±1.31) **	32.1 (±1.79) **
	CCR6^−/−^	86 (±17.2) **	16.0 (±0.79) **	23.9 (±0.76) **	35.4 (±0.83) **
NPs	Naïve	6.9 (±1.1)	1.65 (±0.03)	3.18 (±0.03)	0.94 (±0.03)
	C57BL/6	25.7 (±0.8)	6.03 (±0.02)	16.2 (±0.03)	3.21 (±0.01)
	CCR5^−/−^	10.8 (±0.43) **	2.70 (±0.01) **	4.34 (±0.01) **	1.43 (±0.02) *
	CCR6^−/−^	11.1 (±0.92) **	2.77 (±0.02) **	5.29 (±0.05) **	1.48 (±0.03) *
SMGs	Naïve	17.8 (±2.48)	2.19 (±0.03)	6.31 (±0.04)	5.14 (±0.02)
	C57BL/6	65.8 (±10.9)	8.81 (±0.21)	42.9 (±0.68)	14.1 (±0.38)
	CCR5^−/−^	29.6 (±2.58) **	4.44 (±0.03) **	12.4 (±0.01) **	7.22 (±0.05) **
	CCR6^−/−^	25.5 (±2.19) **	3.69 (±0.03) **	12.9 (±0.16) **	5.52 (±0.16) **
Spleen	Naïve	144 (±30)	16.6 (±0.15)	48.2 (±0.51)	61.2 (±0.63)
	C57BL/6	384 (±52.3)	51.9 (±2.25)	166 (±1.99)	158 (±1.41)
	CCR5^−/−^	189 (±33.4) **	20.2 (±0.30) **	72.3 (±0.77) **	76.5 (±1.04) **
	CCR6^−/−^	158 (±46.6) **	18.1 (±1.35) **	57.2 (±1.26) **	65.8 (±1.77) **

a Mice were nasally immunized weekly for three consecutive weeks with OVA plus Ad-FL.

One week after the last immunization, mononuclear cells from NALT, CLNs, NPs, SMGs and spleen were stained with a combination of the respective mAbs and subjected to flow cytometry analysis by FACSCalibur®.

b Mononuclear cells were stained with PE- labeled anti-CD11c mAb.

c Mononuclear cells were stained with FITC-conjugated anti-CD11b, PE-labeled anti-CD11c, allophycocyanin-tagged anti-B220 and biotinylated anti-CD8 mAbs followed by PerCP-Cy5.5-streptavidin.

d The values shown are the mean ± SEM of five independent experiments. Each experimental group consisted of five mice.

* *p*<0.05,

** *p*<0.01 when compared with C57BL/6 mice nasally immunized with OVA plus Ad-FL.

### CCR5^−/−^ and CCR6^−/−^ Mice Immunized with OVA plus Ad-FL Fail to Undergo OVA-specific IgA Ab Responses

**Figure 4:**
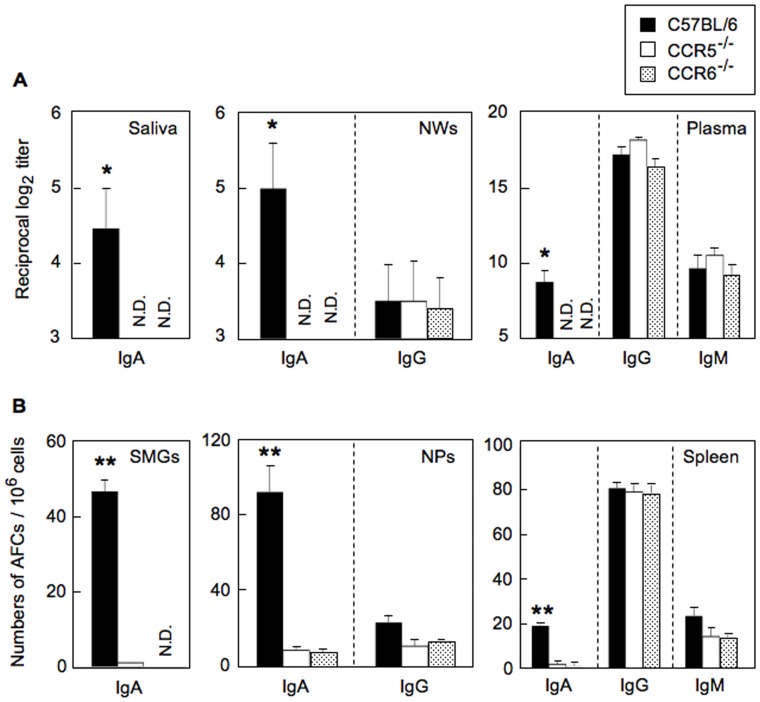
OVA-specific Ab responses in external secretions, plasma and AFC responses in mucosa-associated lymphoid tissues of nasally immunized normal, CCR5^−/−^ or CCR6^−/−^ C57BL/6 mice. Each mouse group was nasally immunized three times at weekly intervals with OVA plus Ad-FL. (A) Seven days after the last immunization, levels of SIgA anti-OVA Abs in saliva, SIgA and IgG Abs in NWs, and IgA, IgG and IgM Abs in plasma were determined by OVA-specific ELISA. (B) MNCs from NPs, SMGs and spleens were isolated 7 days after the last immunization and subjected to an OVA-specific ELISPOT assay to determine the numbers of IgA, IgG and IgM AFCs. The values shown are the mean ± SEM taken from five separate experiments with a total of 25 mice in each experimental group. The ELISA and ELISPOT data for spleen represented the Ab responses from 25 individual mice. MNCs from SMGs and NPs were pooled from 2 or 3 mice and subjected to OVA-specific ELISPOT assay. N.D. means that O.D. values were not detected. *p<0.05, **p<0.01 when compared with normal C57BL/6 mice nasally immunized with OVA plus Ad-FL.

We next assessed the effects of loss of CCR5 and CCR6 expression on OVA-specific Ab responses after nasal immunization with OVA plus Ad-FL. Immune responses were analyzed in plasma, NWs and saliva one week after the last immunization. Loss of neither CCR5 nor CCR6 had an effect on plasma IgG or IgM, or IgG anti-OVA Abs in NWs; however, IgA anti-OVA Ab responses were abrogated in both plasma and external secretions (Figure 4A). Nasal immunization with OVA plus Ad-Luc resulted in essentially no anti-OVA IgA Ab responses in either CCR5^−/−^ or CCR6^−/−^ mice or in normal C57BL/6 mice (data not shown). These findings were also supported at the cellular level, where OVA-specific IgM and IgG AFCs were essentially identical in C57BL/6, CCR5^−/−^ and CCR6^−/−^ mice (Figure 4B). In marked contrast, the numbers of OVA-specific IgA AFCs were essentially absent in spleen, SMGs and NPs of both CCR5^−/−^ and CCR6^−/−^ mice given nasal OVA plus Ad-FL when compared with identically immunized, normal C57BL/6 mice (Figure 4B). Despite the lack of OVA-specific IgA Ab responses in CCR5^−/−^ and CCR6^−/−^ mice, these mice maintained normal levels of total IgA levels in both plasma, nasal washes and saliva (data not shown). These results clearly show that both CCR5 and CCR6 are essential molecules involved in the selective induction of Ag-specific mucosal SIgA and plasma IgA Ab responses. However, it does not distinguish the specific cell types which contribute to IgA-mediated mucosal and systemic immune responses.

### Potential Roles of CCR5/CCR6 Double Positive DCs for the Induction of OVA-specific IgA Ab Responses

**Figure 5:**
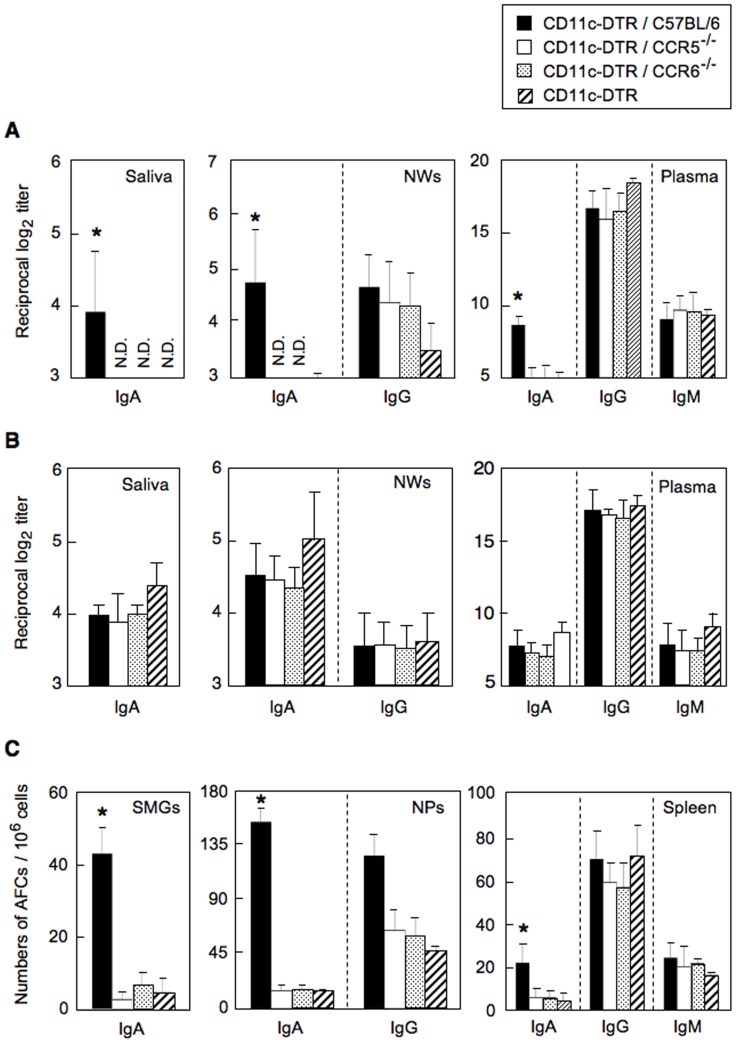
OVA-specific Ab responses in systemic and mucosa-associated lymphoid tissues of chimera mice which lack CCR5- or CCR6-expressing DCs (Figure S1). (A and C) CD11c-DTR, CD11c-DTR/C57BL/6, CD11c-DTR/CCR5^−/−^ and CD11c-DTR/CCR6^−/−^ mice were injected with DT via the intraperitoneal route 6 h before each nasal immunization (three times at weekly intervals with OVA plus Ad-FL). CD11c-DTR/C57BL/6 chimera mice given the DT injection served as positive controls. (B) CD11c-DTR/C57BL/6, CD11c-DTR/CCR5^−/−^, CD11c-DTR/CCR6^−/−^ and normal C57BL/6 were nasally immunized three times at weekly intervals with OVA plus Ad-FL without DT injection. (A and B) Seven days after the last immunization, levels of SIgA anti-OVA Abs in saliva, SIgA and IgG Abs in NWs, and IgA, IgG and IgM Abs in plasma were determined by OVA-specific ELISA. (C) MNCs from NPs, SMGs and spleens were isolated 7 days after the last immunization and subjected to OVA-specific ELISPOT assay to determine the numbers of IgA, IgG and IgM AFCs. The values shown are the mean ± SEM taken from five separate experiments with a total of 25 mice in each experimental group. The data for controls were obtained from two separate experiments which consisted of 6 mice for each experiment. The ELISA and ELISPOT data for spleen represented the Ab responses from 12 individual mice for controls and 25 individual mice for experimental groups. MNCs from SMGs and NPs were pooled from 2 or 3 mice and subjected to OVA-specific ELISPOT assays. N.D. means that O.D. values were not detected. (A and C) *p<0.05 when compared with DT treated, CD11c-DTR/C57BL/6 chimera mice nasally immunized with OVA plus Ad-FL. (B) No significant differences were detected when compared with normal C57BL/6 mice.

The increased expression of CCR5 and CCR6 by CD11c^+^ DCs in nasal-oral mucosal lymphoid tissues certainly implicate this cell type in supporting IgA isotype-specific Ab responses; however direct proof is lacking. In order to more directly probe the role of CCR5^+^ and/or CCR6^+^ DCs in mucosal immunity, CD11c-DTR and normal bone marrow chimeras (CD11c-DTR/C57BL/6), CD11c-DTR and CCR5^−/−^ chimeras (CD11c-DTR/CCR5^−/−^) or CD11c-DTR and CCR6^−/−^ chimeras (CD11c-DTR/CCR6^−/−^) as well as CD11c-DTR mice were employed (Figure S1). When CD11c-DTR mice, CD11c-DTR/CCR5^−/−^ and CD11c-DTR/CCR6^−/−^chimera mice were treated with DT 6 h prior to nasal immunization with OVA plus Ad-FL, whole DCs, CCR5- or CCR6-expressing DCs were transiently depleted in mice; however, these mice contained intact CCR5- or CCR6-expressing T cells. Chimera mice containing normal C57BL/6 bone marrow cells (CD11c-DTR/C57BL/6) with DT treatment were employed as a positive control group. CD11c-DTR, CD11c-DTR/CCR5^−/−^ and CD11c-DTR/CCR6^−/−^ mice all lacked OVA-specific SIgA Ab responses in saliva, NWs or plasma when compared with those responses seen in controls (Figure 5A). It is important to note that all chimera mouse groups without DT treatment showed essentially the same levels of anti-OVA IgA and IgG Ab responses as normal C57BL/6 mice when they were immunized with OVA plus Ad-FL (Figure 5B). Further, significantly reduced numbers of IgA AFCs were seen in SMGs and NPs of chimera mice or CD11c-DTR mice treated with DT (Figure 5C). In contrast, CD11c-DTR/C57BL/6 mice showed significantly increased levels of OVA-specific IgA Ab responses (Figure 5A and 5C). Similarly, C57BL/6 mice treated weekly with DT injections revealed high levels of anti-OVA IgA Ab responses when mice were nasally immunized with OVA plus Ad-FL (data not shown). The levels of anti-OVA IgM and IgG Ab responses in plasma and AFC responses in spleens from these mice were essentially the same as those seen in control mice (Figure 5A and 5B). These results suggest that CCR5- and CCR6- expressing DCs, notably CCR5/CCR6 double positive DCs, play a key role in the induction of OVA-specific IgA Ab responses when mice were nasally immunized with OVA plus Ad-FL as mucosal adjuvant.

## Discussion

The present study has focused on the specific chemokine receptors which are associated with DCs following nasal delivery of Ag plus the DC-inducing adjuvant Ad-FL. Our findings are the first to show that both CCR5 and CCR6 expression by mucosal DCs (CCR5^+^ CCR6^+^ double positive DCs) are essential molecules for potential DC migration and subsequent induction of mucosal and peripheral IgA Ab responses.

It is well accepted that DCs express multiple chemokine receptors and respond to a variety of chemokine ligands [18], [19]. Thus, chemokine receptors expressed by mucosal DCs play central roles in their relocation within mucosal inductive tissues and their migration into mucosal effector sites [6], [8], [9], [11], [12], [13]. For example, CD11b^+^, CD11c^+^, CD8^−^ immature myeloid-type DCs in Peyer’s patches (PPs) expressed either CCR1 or CCR6 for directing their migration toward the subepithelial dome region [6], [11]. In this regard, CCL20 and CCL9, the ligands for these two receptors, were produced by the follicle-associated epithelium [6], [11]. CCR7-expressing, mature lymphoid-type CD8^+^ CD11c^+^ DCs were preferentially found in the T cell zone of PPs, showing that CCR7 expression is important for the relocation of DCs from the subepithelial area to the parafollicular T cell area [6]. Moreover, it has been shown that CCR7 expression by Ag-processing DCs in the intestinal lamina propria and sublingual mucosa are essential for their migration into MLNs or CLNs, respectively [7], [13]. In contrast, our present study clearly showed essentially no increase in CCR7^+^ CD11b^+^ DCs after nasal delivery of OVA plus Ad-FL. On the other hand, increased numbers of CCR5/CCR6-expressing CD11b^+^ DCs were induced in a time-dependent manner beginning in NALT, next in CLNs followed by mucosal effector tissues. Thus, CCR5-expressing CD11b^+^ DCs were increased in both SMGs and NPs. It is possible that increased numbers of these two subsets of DCs in NPs and SMGs may be attributable to migration of CCR5- and CCR5/CCR6-positive CD11b^+^ DCs to their ligand-expressing tissues. To support this, increased levels of CCL3, CCL4 and CCL5 production in NPs and high levels of CCL5 synthesis in SMGs were seen at day 10, which attracted CCR5-expressing CD11b^+^ DCs at this early time point. On the other hand, higher levels of CCL4 and CCL20 by day 21 in NPs and SMGs (but not in NALT) of mice played a key role leading to the increased numbers of CCR5/CCR6-expressing CD11b^+^ DCs in these tissues. Since both CCR5^−/−^ and CCR6^−/−^ mice showed significantly reduced numbers of CD11b^+^ DCs in NPs and SMGs, it is most likely that both CCR5 and CCR6 expression are essential for NALT DCs to migrate into CCL4- and CCL20-producing mucosal effector tissues. Although it is possible that double positive CCR5^+^/CCR6^+^ DCs can express either receptor even in these knockout conditions and reach mucosal effector sites, marked reductions in the numbers of CD11b^+^ DCs suggest that a deficiency of either CCR5 or CCR6 leads to a loss of double positive CCR5^+^/CCR6^+^ DCs in the SMGs and NPs. Further, it has been shown that CCR6 is a key molecule for the induction and regulation of mucosal immune responses by recruiting DCs as well as T and B cells into mucosal effector tissues where allergic responses, active immunity or chronic inflammation occurs [20], [21], [22]. Thus, the CCR6 molecule is one of the essential homing markers for DC migration into mucosal effector sites. Although roles for CCR5 expression in mucosal homing remain to be elucidated, one recent study has shown that adenovirus-specific CD4^+^ T cells expressed higher levels of CCR5 which enhanced their migration to the gut mucosa [23]. Furthermore, others showed that CCR5 plays a role in DC recruitment in Flt3-L-treated mice [24]. Based upon these studies, it is likely that Ad-FL as nasal adjuvant preferentially induced CCR5 expression which served as a mucosal effector tissue-homing molecule. To this end, we are currently testing whether single positive, CCR5^+^/CCR6^−^ and CCR5^−/^CCR6^+^ DC subsets differentiate into the double positive DC subset.

Loss of OVA-specific IgA Ab responses in CCR5^−/−^ and CCR6^−/−^ mice indicates that DCs expressing both CCR5 and CCR6 are key players for the induction of upper respiratory tract mucosal SIgA Ab responses when Ag is delivered by the nasal route. However, it is still possible that lack of mucosal immune responses in CCR6^−/−^ mice is due to the nature of this mutant mouse strain. In this regard, CCR6 is selectively expressed by memory T cells, B cells and DCs, and appears to be involved in the initiation of memory immune responses. Further, CCR6^−/−^ mice have been shown to either lack or have reduced numbers of CD11b^+^ DCs in the subepithelial dome region of PPs [5], [25]. Indeed, our findings showed that this DC population was reduced in the oral-nasopharyngeal mucosal tissues as well. In addition, other studies reported that orally immunized CCR6^−/−^ mice fail to support Ag-specific SIgA Ab responses [5], [26]. Furthermore, CCR6^−/−^ mice showed impaired M cell development, a cell type that plays a key role in Ag uptake for the induction of mucosal immunity. In contrast to CCR6^−/−^ mice, CCR5^−/−^ mice have intact CD11b^+^ DCs and normal M cell development. However, it has been reported that CC-chemokines differentially enhanced mucosal and systemic Ab responses. Thus, CCL4 as nasal adjuvant resulted in selective upregulation of Ag-specific SIgA but not IgG and IgM Ab responses [27]. Taken together, these results indicate that CCR5 expression by nasal DCs is essential both for the increased numbers of DCs and for their support of Ag-specific SIgA Ab responses in the upper respiratory tract and oral cavity.

In order to provide more direct roles for CCR5- and CCR6-expressing DCs in the regulation of mucosal IgA immune responses, we constructed chimera mice by mixed bone marrow transplantation from CD11c-DTR and CCR5^−/−^ or CCR6^−/−^ mice. By injecting DT prior to each nasal immunization, these chimera mice became deficient in CCR5- or CCR6-expressing DCs. Impaired OVA-specific mucosal IgA Ab responses were seen in both CD11c-DTR/CCR5 and CD11c-DTR/CCR6 mice. If single positive CCR5 or CCR6 DCs play a role, either CD11c-DTR/CCR5- or CD11c-DTR/CCR6-chimera mice should show intact Ag-specific IgA Ab responses. Further, we showed that CCR5/CCR6 double positive DCs are increased in mucosal inductive and effector tissues after nasal immunization. Since CCR5^+^/CCR6^−^ DCs are also increased in mucosal effector tissues, it is possible that both CCR5^+^/CCR6^−^ and double positive CCR5^+^/CCR6^+^ DC subsets may be required for the induction of mucosal SIgA Ab responses. Nevertheless, our results clearly show that CCR5/CCR6 double positive nasal DCs play an essential role in the induction of OVA-specific IgA Ab responses. In contrast, OVA-specific systemic IgM and IgG Ab responses in these chimera mice were essentially the same as those responses in normal mice given nasal OVA plus Ad-FL. These findings suggest that different types of APCs in the nasal mucosa are responsible for systemic IgM and IgG but not mucosal IgA Ab responses. Indeed, it has been suggested that some DCs in the intestinal lamina propria extend their dendrites between epithelial cell junctions into the lumen for taking-up Ags (intraepithelial DCs) for the initiation of Ag-specific systemic IgG Ab responses [28], [29], [30], [31]. Similar types of intraepithelial DCs were also reported in the upper respiratory tract [32]. In addition, M cells are found in the lungs and on the bronchial bifurcation of the airways. Airway challenge with *Mycobacterium tuberculosis* resulted in the entry of this pathogen via these lung M cells [33]. Further, others have also noted the presence of M cells in the nasal cavity itself which are capable of taking up both particulate and soluble antigens delivered by the nasal route [34]. Thus, not only DCs but also other immune cells in the lamina propria of the nasal cavity play an essential role in the induction of Ag-specific systemic IgM and IgG Ab responses. Another potential explanation for impaired IgA but intact systemic IgM and IgG Ab responses in chimera mice is that DCs must be present when vaccines are administered via the mucosal route. Indeed, DT-treated CD11c-DTR mice given nasal OVA plus Ad-FL resulted in essentially no anti-OVA IgA but intact IgM and IgG Ab responses. The absence of DCs may lead to the majority of Ag and adjuvant transversing into the systemic compartment. Since DC depletion by DT is transient, recovered systemic DCs could handle circulating systemic Ags for induction of IgG Ab responses, whereas mucosal DCs would miss this opportunity to recognize Ag and adjuvant after recovery. To support this, our group has shown that a mucosal DC targeting immunization strategy is essential for the induction of Ag-specific IgA Ab responses in mucosal effector sites [14], [16], [17], [35], [36]. It is also possible that CCR5/CCR6 double positive CD11b^+^ DCs directly regulate Ag-specific IgA Ab responses by inducing sIgA^+^ B cells to differentiate into IgA producing plasma cells. Indeed it has been shown that APRIL-expressing mucosal DCs interact with TACI expressing B cells to induce IgA Ab responses [37], [38]. Further, retinoic acid (RA)-producing mucosal DCs play key roles in mucosal T and B cell migration for the induction of Ag-specific mucosal immunity [39]–[41]. In addition, others showed that follicular DCs (FDCs) in PPs directly induce IgA CSR by PP B cells via RA and BAFF and APRIL pathways [42]. In this regard, we are currently investigating potential cellular and molecular mechanisms whereby CCR5/CCR6 double positive CD11b^+^ DCs express BAFF and APRIL and produce RA for the induction of Ag-specific SIgA Ab responses.

In summary, the present study showed that Ad-FL as a nasal adjuvant induced CD11b^+^ DCs which expressed increased levels of CCR5 and CCR6 for their subsequent migration into CCL3- and CCL20- producing mucosal effector tissues. In this regard, lack of these chemokine receptors failed to allow CD11b^+^ DC recruitment and subsequent induction of Ag-specific SIgA Ab responses in oral-nasopharyengeal effector tissues. Defining the cellular and molecular mechanisms for mucosal DC recruitment will help provide a better understanding of how mucosal immune responses are initiated including immunity, inflammation and tolerance. Our present findings clearly support a strategy for the development of a nasal immunization protocol which recruits DCs for vaccine uptake and their subsequent increase in mucosal effector sites where protective SIgA Ab responses could be induced for host protection.

## Supporting Information

Figure S1 **Preparation of chimera mice by bone marrow transplantation.** C57BL/6 mice were irradiated (1000 rads), and injected intravenously with a mixture of bone marrow cells from CD11c-DTR and C57BL/6 (CD11c-DTR/C57BL/6), CD11c-DTR and CCR5^−/−^ (CD11c-DTR/CCR5^−/−^), or CD11c-DTR and CCR6^−/−^ (CD11c-DTR/CCR6^−/−^) mice 6 h after irradiation. Diphtheria toxin (DT) (100 ng/mouse) was injected into CD11c-DTR/C57BL/6 chimera, CD11c-DTR/CCR5^−/−^ chimera and CD11c-DTR/CCR6^−/−^ chimera mice via the intraperitoneal route 6 h before each nasal immunization.
(TIF)
