# Supplementary figures and images for: Potential Roles of CCR5+ CCR6+ Dendritic Cells Induced by Nasal Ovalbumin plus Flt3 Ligand Expressing Adenovirus for Mucosal IgA Responses
Source: PLoS One. 2013 Apr 2;8(4):e60453. doi: 10.1371/journal.pone.0060453 (PMC3615010; doi:10.1371/journal.pone.0060453)

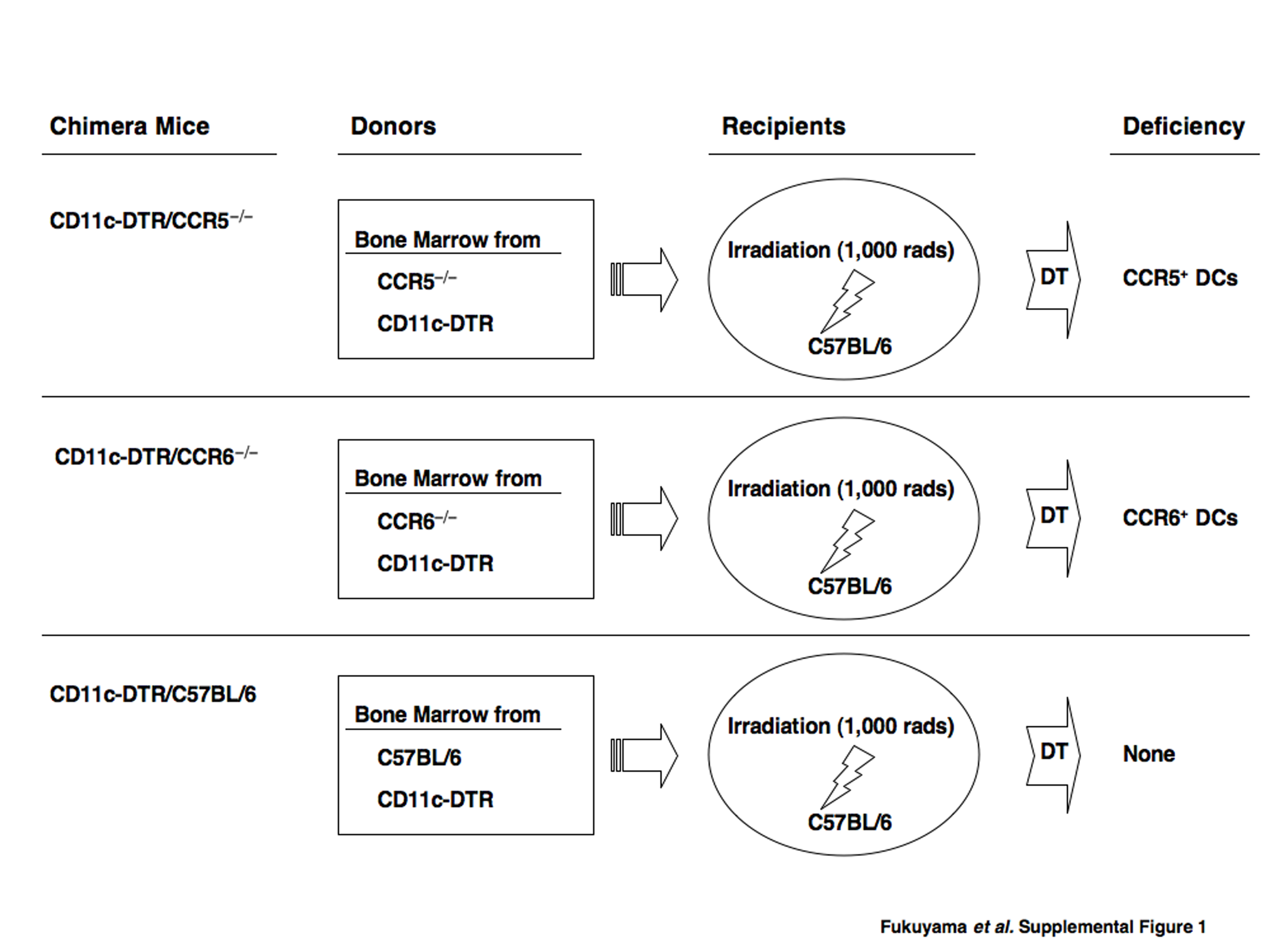

Supplement: Figure S1 — Preparation of chimera mice by bone marrow transplantation. C57BL/6 mice were irradiated (1000 rads), and injected intravenously with a mixture of bone marrow cells from CD11c-DTR and C57BL/6 (CD11c-DTR/C57BL/6), CD11c-DTR and CCR5−/− (CD11c-DTR/CCR5−/−), or CD11c-DTR and CCR6−/− (CD11c-DTR/CCR6−/−) mice 6 h after irradiation. Diphtheria toxin (DT) (100 ng/mouse) was injected into CD11c-DTR/C57BL/6 chimera, CD11c-DTR/CCR5−/− chimera and CD11c-DTR/CCR6−/− chimera mice via the intraperitoneal route 6 h before each nasal immunization. (TIF) [file pone.0060453.s001.tif]
